# A Fatal Case of Capsular Serovar D of Capnocytophaga canimorsus Infection Following a Dog Bite in a Previously Healthy Adult

**DOI:** 10.7759/cureus.104503

**Published:** 2026-03-01

**Authors:** Hiroyuki Tanimoto, Hisashi Dote, Hiroki Shinozaki, Yuuki Meguro, Shoko Ishii, Shimon Aoki, Takeaki Nishibori, Kazuki Kobayashi, Mamoru Miyajima

**Affiliations:** 1 Emergency and Critical Care Medicine, Nagaoka Red Cross Hospital, Nagaoka, JPN; 2 Emergency and Critical Care Medicine, Seirei Hamamatsu General Hospital, Hamamatsu, JPN; 3 Department of Infectious Diseases, Nagaoka Red Cross Hospital, Nagaoka, JPN

**Keywords:** acute infectious purpura fulminans, capnocytophaga canimorsus, capsular serovar d, disseminated intravascular coagulation, dog bite, hyposplenism, septic shock

## Abstract

We report a rapidly fatal case of *Capnocytophaga ​​​​canimorsus* septic shock with suspected acute infectious purpura fulminans (AIPF) in a 61-year-old man following a dog bite to the finger. Although he appeared previously healthy, retrospective analysis revealed a markedly small spleen (54 mm in diameter) on computed tomography and Howell-Jolly bodies on a peripheral blood smear, indicating unrecognized functional hyposplenism. He developed systemic symptoms within 48 h and progressed to respiratory failure and refractory shock within 1 h of presentation to the emergency department. The blood smear also demonstrated phagocytosed Gram-negative rods, prompting immediate broad-spectrum antimicrobial therapy and intensive care. However, multiple organ failure progressed, and the patient died on hospital day three. *C. canimorsus* was identified from blood cultures five days after death; capsular serovar D was confirmed subsequently. This case highlights that *C. canimorsus* infection can trigger explosive clinical deterioration, particularly when uncommon virulent strains like serovar D infect patients with unrecognized splenic atrophy.

## Introduction

Dog and cat bites are frequent presentations in emergency departments, typically resulting in localized soft-tissue infections. However, invasive infections caused by animal oral flora can rapidly progress to severe systemic disease [1]. *Capnocytophaga canimorsus*, a Gram-negative bacillus and normal oral commensal in dogs and cats, is a rare but recognized cause of fulminant sepsis following bites or close contact [2]. Although the incidence of *C. canimorsus *infection is extremely low, estimated at 0.5-0.67 cases per million population [3], the mortality rate is remarkably high, ranging from 26% to 30% [4]. The virulence of the organism is significantly influenced by its polysaccharide capsule (capsular serovars), which facilitates immune evasion and resistance to macrophage phagocytosis [5].

Severe disease is predominantly associated with immunocompromised states, including a post-splenectomy state, cirrhosis, diabetes mellitus, alcohol use disorder, and steroid exposure [6-10]. Nevertheless, in both immunocompromised and seemingly immunocompetent individuals, *C. canimorsus *sepsis can be complicated by acute infectious purpura fulminans (AIPF) [11]. AIPF is characterized by rapidly progressive purpura, disseminated intravascular coagulation (DIC), and peripheral ischemia, carrying an exceptionally poor prognosis.

We present a hyperacute fatal case of *C. canimorsus *septic shock complicated by suspected AIPF following a dog bite. Notably, the patient had no known immunocompromising conditions prior to admission but was subsequently found to have occult functional hyposplenism (splenic atrophy). In this report, we aimed to highlight the synergistic impact of this unrecognized host vulnerability and the uncommon, highly virulent capsular serovar D on the patient's fulminant deterioration.

## Case presentation

Patient information and history

A 61-year-old man with hypertension treated with amlodipine (5 mg daily) was bitten on the right index finger by his pet dog. He had been bitten by the same dog multiple times previously without complication and had never sought medical care. After the current bite, he did not perform wound irrigation and continued cooking immediately. The next day, he developed a sore throat and shaking chills. On day two, he developed fever, generalized limb pain, low back pain, and vomiting; he was transported to the emergency department via ambulance.

Examination on arrival

**Figure 1:**
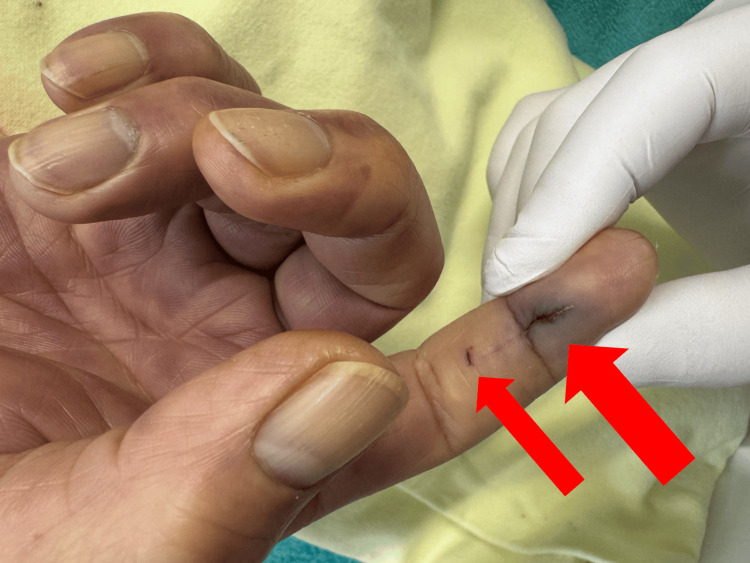
Cutaneous findings of the dog bite on the right index finger. Two small puncture wounds (arrows) are visible near the distal interphalangeal joint of the right index finger.

On arrival, the patient was alert and oriented (Glasgow Coma Scale E4V5M6). Vital signs were as follows: temperature 37.5°C, respiratory rate 30 breaths/min, heart rate 98 beats/min, blood pressure 117/79 mmHg, and oxygen saturation 96% on room air. Lung auscultation was unremarkable. On the right index finger, two puncture wounds were present near the distal interphalangeal joint with mild erythema and swelling but no purulent drainage (Figure 1). Purpuric skin lesions were minimal at presentation but expanded rapidly during resuscitation.

Laboratory and imaging studies

Initial laboratory testing demonstrated leukopenia (white blood cell count 1,100/µL), elevated C-reactive protein (13.66 mg/dL), and markedly elevated procalcitonin (83.05 ng/mL). Other admission laboratory values, including arterial blood gas analysis, are summarized in Table 1.

**Table 1:** Longitudinal laboratory and arterial blood gas findings from arrival to the terminal phase. This table demonstrates the fulminant progression of multiple organ failure. MV: mechanical ventilation; FDP: fibrin/fibrinogen degradation products; WBC: white blood cell count; PT-INR: prothrombin time-international normalized ratio; aPTT: activated partial thromboplastin time

Parameters (units)	Ref. range	0 h (arrival)	8 h (worsening)	24 h (terminal)
Arterial blood gas				
pH	7.35-7.45	7.352	7.026	7.087
PaCO_2_ (mmHg)	35.0-45.0	29.5	29.3	34.1
PaO_2_ (mmHg)	80.0-100.0	101.4	98.7	67.2
HCO_3_- (mmol/L)	22.0-26.0	16	7.5	10
Base excess (mmol/L)	-2.0 to +2.0	-8.5	-21.4	-16.9
PaO_2_/FiO_2_ ratio	>400	253.5	98.7	67.2
Lactate (mmol/L)	0.5-2.2	8.5	16.6	28.5
Oxygen/ventilation	-	Mask 5L/min	MV	MV
FiO_2_	-	0.4	1.0	1.0
Hematology				
WBC (/µL)	3300-8600	1100	1900	2920
Platelets (×10^4^/µL)	15.8-34.8	1.4	3.2	1.3
Coagulation				
PT-INR	0.90-1.10	2.39	2.63	>15
aPTT (s)	25.0-40.0	>400	>400	>400
FDP (µg/mL)	<5.0	324.8	-	272.3
Biochemistry				
Glucose (mg/dL)	70-109	45	9	213
Creatinine (mg/dL)	0.65-1.07	2.13	2.37	2.53
Procalcitonin (ng/mL)	<0.05	83.05	-	-

**Figure 2:**
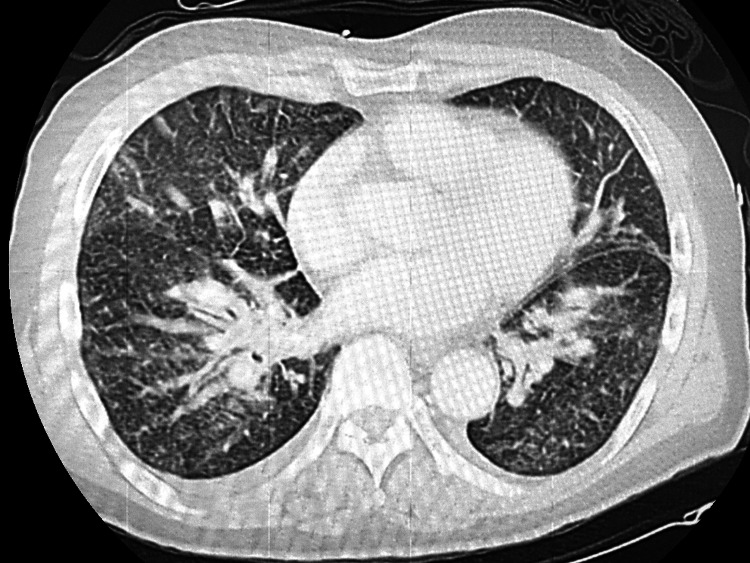
Computed tomography (CT) of the chest on admission. Contrast-enhanced axial image of the chest demonstrating increased pulmonary vascular markings and mild peribronchovascular thickening.

**Figure 3:**
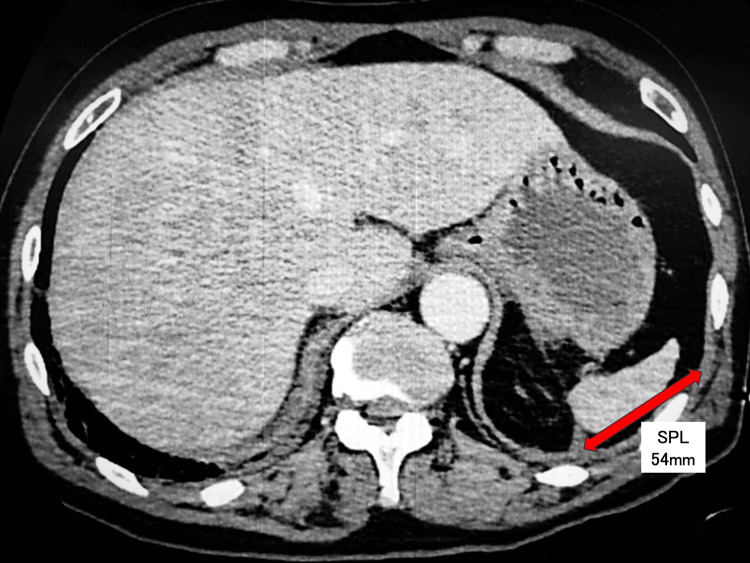
Abdominal computed tomography (CT) showing splenic atrophy. Axial view of the upper abdomen showing the spleen (SPL). The spleen is markedly small, with a maximum diameter of 54 mm (arrow). This finding indicates splenic atrophy.

Computed tomography (CT) of the chest showed increased pulmonary vascular markings (Figure 2). Abdominal CT revealed no focal source of infection; however, the spleen was notably small, measuring approximately 54 mm in maximum diameter (normal adult range: 90-120 mm) (Figure 3) [12]. A peripheral blood smear revealed neutrophils containing phagocytosed Gram-negative rods, raising suspicion for Capnocytophaga species, given the history of a dog bite.

Treatment and clinical course

**Figure 4:**
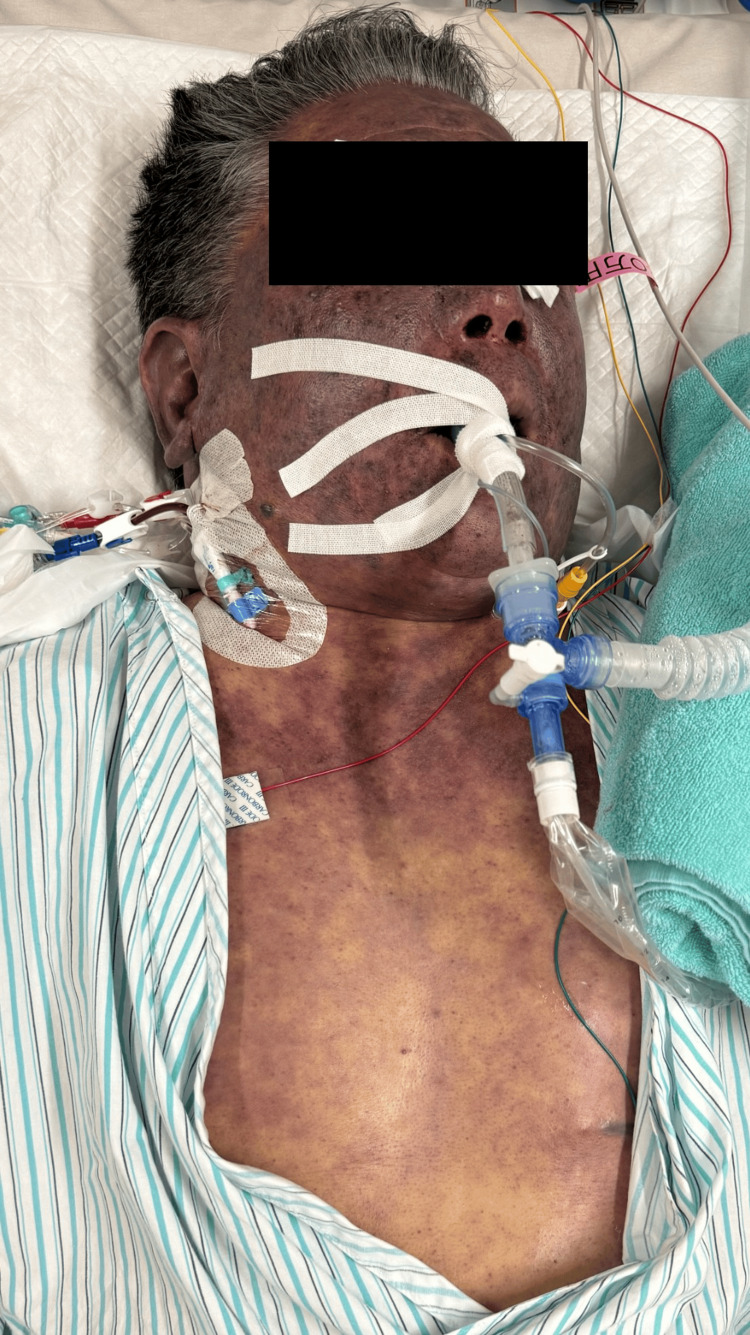
Clinical manifestation of acute infectious purpura fulminans (AIPF). Clinical photograph taken during the intensive care unit stay. Extensive, confluent purpuric patches and ecchymosis are visible across the trunk and extremities. The rapid expansion of these lesions within hours of admission is a hallmark of the explosive disseminated intravascular coagulation (DIC) associated with *Capnocytophaga canimorsus *infection.

The patient was initially conversant; however, within one hour of arrival, he developed rapidly worsening respiratory failure and hypotension. He was intubated and mechanically ventilated (FiO_2_: 1.0), but his PaO_2_/FiO_2_ ratio dropped precipitously to 98.7 within 8 h, indicating severe acute respiratory distress syndrome (ARDS). Purpuric lesions expanded quickly over the extremities, supporting the suspicion of AIPF (Figure 4).

Given the hyperacute presentation of undifferentiated septic shock with rapidly expanding purpura, an ultra-broad-spectrum empiric antimicrobial regimen was initiated immediately - intravenous meropenem, clindamycin, and azithromycin. Meropenem was selected for broad Gram-negative and anaerobic coverage. Clindamycin was added for its potential toxin-suppressing effects in the setting of suspected fulminant soft-tissue infection or toxin-mediated shock, while azithromycin was included to ensure coverage of atypical pathogens.

Because shock remained refractory with progressive metabolic acidosis and anuria, continuous renal replacement therapy and polymyxin B hemoperfusion were initiated. Despite maximal support, the patient developed profound metabolic collapse, evidenced by extreme hypoglycemia (9 mg/dL) at 8 h and uncontrollably rising lactate levels (reaching 28.5 mmol/L at 24 h). The patient died of progressive shock and multiple organ failure on hospital day three.

Microbiological diagnosis and autopsy

Blood cultures yielded *C. canimorsus *five days after death. Capsular serovar D was identified by polymerase chain reaction at Japan’s National Institute of Infectious Diseases. Autopsy demonstrated findings consistent with severe sepsis and DIC, including microthrombi in the myocardium and renal glomeruli. Howell-Jolly bodies were noted on the peripheral smear, suggesting previously unrecognized functional hyposplenism secondary to the splenic atrophy observed on CT [13].

## Discussion

This report illustrates three clinical points regarding fulminant *C. canimorsus *sepsis. First, the infection can be rapidly fatal. In this patient, systemic symptoms developed within 48 h of a minor bite, and he progressed to respiratory failure and refractory shock within 1 h of arrival. This tempo is characteristic of fulminant *C. canimorsus* sepsis and necessitates immediate empiric therapy [2,7,8].

Second, the host had an unrecognized predisposition - splenic atrophy. While initially considered immunocompetent, the patient's CT revealed a significantly small spleen (maximum diameter 54 mm; normal adult range: 90-120 mm [12]), and peripheral smears showed Howell-Jolly bodies [13]. Because the spleen is central to the opsonization and clearance of encapsulated organisms, this anatomical atrophy and resultant functional hyposplenism likely increased susceptibility to pathogens and contributed to manifestations such as suspected AIPF [11]. Splenic dysfunction may not be clinically apparent before such catastrophic infections.

Third, the pathogen was identified as capsular serovar D, an uncommon but potentially highly virulent strain in susceptible hosts. While capsular serovars A, B, and C are responsible for the vast majority of severe *C. canimorsus *infections globally due to their high human virulence [14], serovar D is rarely encountered in human disease. However, the emergence of fatal cases associated with serovar D, including a previously reported case in Japan characterized by fulminant sepsis [15], suggests that this specific serovar possesses a high level of virulence when synergistic with host vulnerabilities such as hyposplenism. Our case further reinforces this observation; the patient developed suspected AIPF and profound metabolic collapse despite intensive intervention. The extreme hypoglycemia (9 mg/dL) and severe lactatemia (28.5 mmol/L) observed are extraordinary findings that likely reflect severe metabolic dysregulation driven by a combination of hepatic dysfunction impairing gluconeogenesis, cytokine-mediated metabolic failure, overwhelming endotoxemia, and relative adrenal insufficiency. Clinicians must recognize that the rarity of a serovar does not equate to attenuated toxicity.

From a diagnostic standpoint, peripheral blood smear findings can provide early clues. Molecular diagnostics such as 16S rRNA PCR may be considered when cultures remain negative [10,15]. Regarding therapy, early empiric antibiotics and aggressive supportive care based on international guidelines are critical. In the emergency setting, given the hyperacute presentation of undifferentiated septic shock and rapidly progressive purpura, an ultra-broad-spectrum empiric regimen was initiated. Meropenem provided extensive Gram-negative and anaerobic coverage, including Capnocytophaga species [16]. Following skin and soft-tissue infection guidelines, clindamycin was specifically added for its toxin-suppressing effects to cover potential toxic shock syndrome or necrotizing fasciitis [17]. Additionally, azithromycin was included to cover atypical respiratory pathogens given the precipitous onset of severe ARDS [18].

## Conclusions

In patients who develop systemic symptoms after a dog or cat bite, severe *Capnocytophaga canimorsus *infection can occur even when no immunocompromising condition has been previously identified. A small spleen on imaging may indicate unrecognized hyposplenism and should prompt heightened concern for fulminant disease, including suspected acute infectious purpura fulminans (AIPF). Furthermore, while rare, infections associated with uncommon capsular types, such as serovar D, can be exceptionally severe when combined with such host vulnerabilities. In the emergency department, clinicians should anticipate rapid deterioration, triage these patients as high risk, and initiate empiric broad-spectrum antimicrobial therapy and supportive care without delay.
